# Crystalline Forms of Trazodone Dihydrates

**DOI:** 10.3390/molecules26175361

**Published:** 2021-09-03

**Authors:** M. John Plater, William T. A. Harrison

**Affiliations:** Department of Chemistry, University of Aberdeen, Meston Walk, Aberdeen AB24 3UE, UK; w.harrison@abdn.ac.uk

**Keywords:** trazodone, crystalline hydrate, polymorph, crystallography

## Abstract

In this study, treatment of anhydrous trazodone powder with ammonium carbamate in warm water crystallised two new polymorphs or dihydrates of trazodone after 5 h, whose structures were determined by X-ray single crystal diffraction. Each dihydrate contains infinite zigzag hydrogen-bonded chains of water molecules, which are stabilised by the N4 acceptor atom of the piperazine ring and the pendant carbonyl O1 atom of the triazole ring, as well as other water molecules. The strong dipole moment expected for the O1 atom makes it a good hydrogen bond acceptor for stabilising the chains of water molecules. Each molecule of trazodone has a similar conformation in both hydrates, except for the propyl chains, which adopt different conformations: anti-gauche in the β hydrate (triazole N-C-C-C and C-C-C-piperazine N) and anti-anti in the γ hydrate. Both piperazine rings adopt chair conformations, and the *exo*cyclic N-C bonds are in equatorial orientations. The Hirshfeld surfaces and two-dimensional fingerprint plots for the polymorphs were calculated using CrystalExplorer17, which indicated contacts significantly shorter than the sum of the van der Waals radii in the vicinity of the piperazine N4 and triazole O1 atoms corresponding to the strong hydrogen bonds accepted by these atoms.

## 1. Introduction

Water, ubiquitous for the presence of life, can have important and sometimes adverse effects on the manufacture of drugs and their performance. It can be introduced through an active ingredient, excipients, or the atmosphere, and can increase interactions between a drug and its excipients, dissolve soluble components or induce phase transitions [1]. The absorption of moisture can affect the properties of the powders used in pharmaceutical processing, such as compaction, blending, bulk density and flow [2,3,4,5]. For some drugs taken as oral solids, absorbed water can affect dissolution, disintegration and chemical stability [6,7,8,9,10]. Water has a wide-ranging impact on physical and chemical properties and must therefore be accounted for at all stages of the drug substance and product manufacture as well as the shelf life of the product. The hygroscopicity, a measure of the water vapour taken up by a solid, which might affect its surface and bulk properties, is an important criterion for deciding the solid-state form of the oral drug [11]. The moisture absorption of the excipients, such as binding agents and disintegrants, are also evaluated so that in combination with the drug, the product is stable during processing and storage of the drug.

When a compound or a drug co-crystallises with water, a new crystalline phase, termed a hydrate, is formed. Hydrates are a subset of a larger class of crystalline solids, called solvates, which have the solvent of crystallisation included in the structure. Hydrates may form by crystallisation from solution or by the uptake of water vapour in the solid state. An example is metacetamol, an isomer of paracetamol, which forms a hemihydrate [12]. Metacetamol is also of interest because it can form two anhydrous polymorphs from aqueous solution if the crystallisation conditions are precisely controlled [13]. Compounds may form multiple hydrates with the same stoichiometry, known as hydrate polymorphs [14], or with different amounts of water. Hydrates are distinct thermodynamic phases with different properties, so control of the product performance requires control of the hydrate appearance or disappearance. Thermal, spectroscopic and diffraction techniques are used to characterise hygroscopicity and hydration states [14,15,16,17,18]. Gravimetric water sorption (GVS) is also used for characterising hygroscopic behaviour in pharmaceutical drugs.

**Figure 1:**
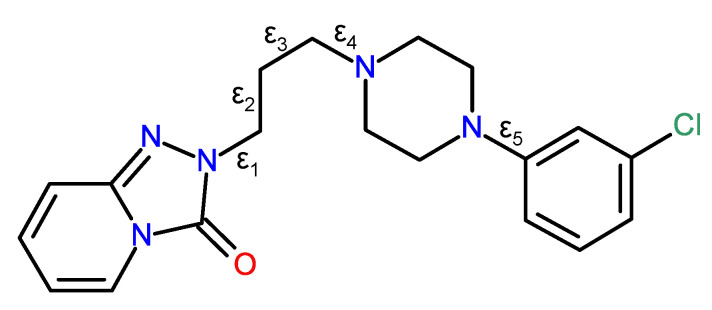
Chemical structure of trazodone with key torsion angles indicated.

Trazodone, C_19_H_22_ClN_5_O, systematic name 2-{3-[4-(3-chlorophenyl)piperazin-1-yl]propyl}[1,2,4] triazolo[4,3-*a*]pyridine-3(2*H*)-one, is an antidepressant medication discovered in the 1960s and now widely prescribed throughout the world [19,20]. The crystal structures of a number of molecular salts containing the C_19_H_23_ClN_5_O^+^ trazadonium cation with different counter ions have been reported, including with chloride ions (Cambridge Structural Database [21] refcode CPTAZP) [22], 2,4-dinitrophenolate (DOZMIR) [23], nitrate (FIRHAS) [23], thiocyanate (FIRHEW) [24], tetrafluoroborate (FIRHIA) [24], iodide (ZEXPEA) [25] and *oxalate decahydrate* (ZEXREC) [25]. In every case, the N atom of the piperazine ring adjacent to the propyl chain (see Figure 1) is protonated and the N^+^-H grouping thereby formed participates in a charge-assisted N-H···X hydrogen bond to the anion.

So far as we are aware, the solid-state structure of neutral trazodone has not yet been described, but the crystal structure of a dihydrate of trazodone was recently reported [25] as part of a study into the high-throughput nano-crystallisation of organic salts: its unit cell is monoclinic, space group *P*2_1_/*c* (Cambridge Structural Database refcode ZEXPAW), and it is hereafter designated as the α polymorph of C_19_H_22_ClN_5_O·2H_2_O. This paper reports the syntheses and characterisation of two new dihydrates of trazodone (the β and γ polymorphs) by X-ray single-crystal structure determination and a structural comparison of the three polymorphs.

## 2. Results and Discussion

### 2.1. Structure of β-trazodone Dihydrate

**Figure 2:**
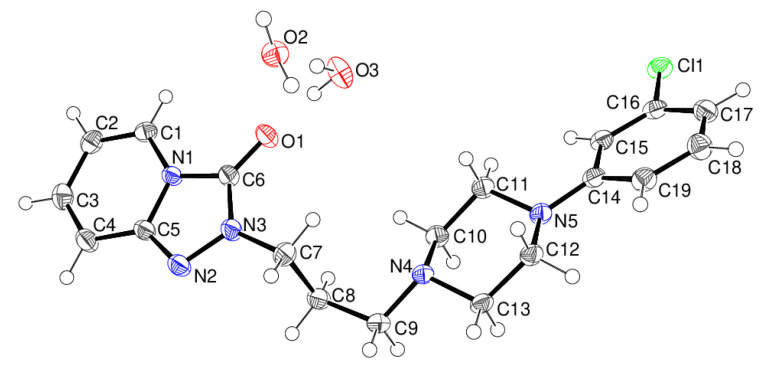
The molecular structure of β-C_19_H_22_ClN_5_O·2H_2_O showing 50% displacement ellipsoids.

The preparation details are described in the experimental section. The air-dried hydrate showed good microcrystalline stability after many weeks by examination under a microscope. The two morphologies of laths and blocks appeared the same. The asymmetric unit of β-trazodone dihydrate contains one trazodone molecule and two water molecules (Figure 2): the β polymorph crystallises in *P*2_1_/*n*, an alternative setting of the *P*2_1_/*c* space group of the α polymorph but it is clearly different, as discussed below. The piperazine ring adopts a normal chair conformation with the exocyclic N4-C9 and N5-C14 bonds in equatorial orientations, and the dihedral angle between the N_2_C_4_ ring (all atoms) and the adjacent C14-C19 benzene ring is 17.67 (7)°. Otherwise, the bond lengths and angles in β-C_19_H_22_ClN_5_O·2H_2_O fall within their usual ranges and the key torsion angles defined in Figure 1 are discussed below.

**Figure 3:**
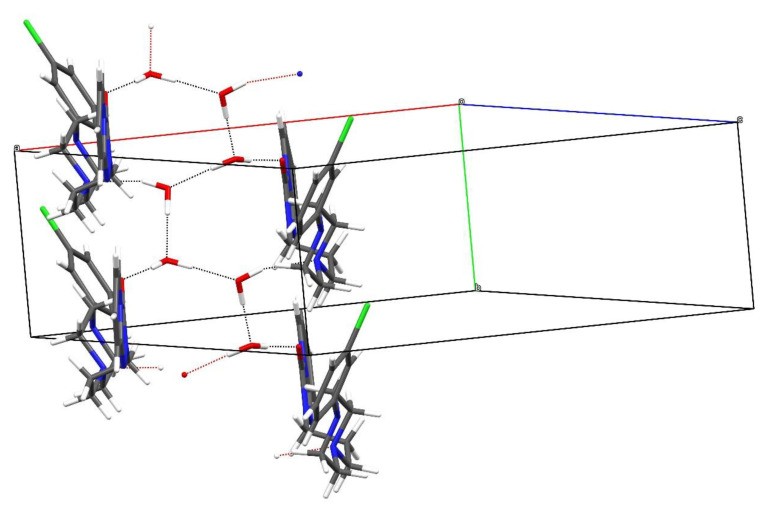
Hydrogen-bond network in β-C_19_H_22_ClN_5_O·2H_2_O showing the formation of [010] zigzag chains of water molecules. The first water molecule forms an O-H...O hydrogen bond to the other water molecule and an O-H...N link to an adjacent trazodone molecule; the second water molecule forms an O-H...O_w_ (w = water) link to the first water molecule and an O-H...O bond to a trazodone O atom.

In the extended structure of β-C_19_H_22_ClN_5_O·2H_2_O, O-H···O and O-H···N hydrogen bonds arising from the water molecules link the constituent species (Supplementary Table S1). The acceptor atoms are the N4 atom of the piperazine ring, the pendant carbonyl O1 atom of the triazole ring, as well as other water molecules, which results in infinite zigzag hydrogen-bonded chains propagating in the [010] direction (Figure 3), and weak C-H···N, C-H···O and C-H···Cl hydrogen bonds may help to consolidate the structure.

### 2.2. Structure of γ-trazodone Dihydrate

**Figure 4:**
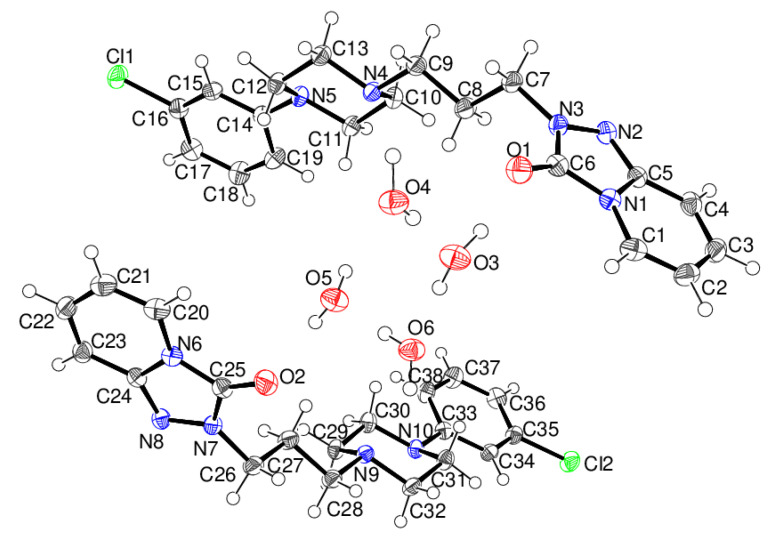
The molecular structure of γ-C_19_H_22_ClN_5_O·2H_2_O showing 50% displacement ellipsoids.

The asymmetric unit of γ-trazodone dihydrate contains two organic molecules and four water molecules, i.e., *Z*′ = 2. The first trazodone molecule contains atoms C1–C19, N1–N5, O1 and Cl1, and the second molecule contains atoms C20–C38, N6–N10, O2 and Cl2 (Figure 4).

The conformations of the C1- and C20-containing trazodone molecules are very similar (r.m.s. overlay fit = 0.044 Å), and the structure of γ-C_19_H_22_ClN_5_O·2H_2_O indeed shows strong pseudo-symmetry with respect to space group *Pbca*, where these two organic molecules and pairs of water molecules would be related by crystallographic inversion symmetry. The presence of local (non-crystallographic) centres of symmetry is a common feature [26] of space group *Pca*2_1_, and refinements in *Pbca* gave much poorer fits (*R*(*F*) ~ 0.20) and unrealistic displacement parameters; we take merohedrally-twinned *Pca*2_1_ to be correct in this case. Both piperazine rings adopt chair conformations, and all the *exo*cyclic N-C bonds are in equatorial orientations, and the dihedral angles between the piperazine and benzene rings are 23.14 (13) and 22.57 (13)° for the C1- and C20-molecules, respectively.

**Figure 5:**
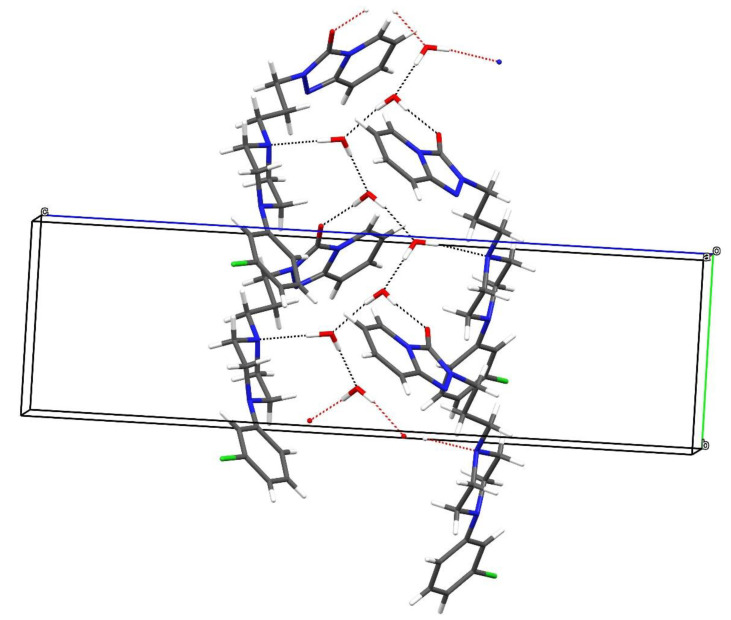
Hydrogen bonds in γ-C_19_H_22_ClN_5_O·2H_2_O, which generate [010] chains of water molecules.

Hydrogen-bond geometrical data for the γ polymorph are listed in Supplementary Table S2, where it may be noted that the same trazodone atoms as in the β polymorph, viz., the N atoms adjacent to the propyl chain and the triazole-ring O atoms are the acceptors for the strong O-H···X links. Once again, the packing is consolidated by several weak C-H···O, C-H···N and C-H···Cl interactions but given their H···X lengths, these are presumably very weak. The hydrogen-bonding pattern arising from the classical O-H···X (X = N, O) bonds is shown in Figure 5, which reveals a rather similar zigzag topology to the equivalent chain in the β polymorph.

### 2.3. Comparison of the Three Polymorphs

As might be expected, the geometries of the rigid triazole–pyridine fused ring system, the chair-like piperazine ring, and the chlorobenzene ring barely differ between the polymorphs, and the key differences between the structures are associated with different torsion angles in the trazodone molecules, above all those associated with the conformation of the propyl chain (Table 1; compare Figure 1). The α-polymorph [25] shows disorder of the fused ring system and propyl chain over two ‘flipped’ orientations, and the torsion angles stated in Table 1 refer to the major disorder (occupancy = 0.90) component and were calculated with PLATON [27].

**Figure 6:**
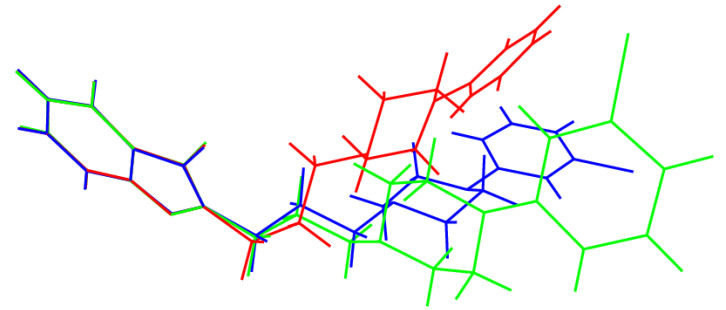
Overlay plot of the trazodone molecules in the α (red), β (green) and γ (blue) polymorphs with the fused ring systems superimposed.

It may be seen from the ε_2_ and ε_3_ torsion angles listed in Table 1 that the propyl chains in these polymorphs adopt three different conformations, namely gauche-anti (working from the fused ring system to the piperazine ring) for α, anti-gauche for β and anti-anti for γ. This results in the molecules having very different overall conformations, as shown in the overlay plot generated with QMOL [28] in Figure 6. The anti-anti conformation of the propyl chain for the γ polymorph is probably the most stable conformation because the bulky groups of the trazodone molecule are furthest apart. In the gauche-anti and the anti-gauche conformations for the α and β polymorphs, respectively, there is more steric interaction between the gauche groups. A possible gauche-gauche polymorph has not been observed because it would be less stable. We may speculate that for the α and β polymorphs, crystal packing forces and hydrogen bonds are leading to a less stable conformation of the propyl chain in the gauche-anti and the anti-gauche conformations, compared to that of the anti-anti conformation of the propyl chain for the γ polymorph. The ε_1_ torsion angles are similar for the three polymorphs, and these presumably reduce steric repulsion between O1 and C8 and its attached H atoms (O···H ≈ 3.5 Å). The ε_4_ angles have similar magnitudes (and a change of sign for the α polymorph) and again reflect the minimisation of steric factors about the C9-N4 bond by way of a gauche conformation. Finally, ε_5_ represents the optimisation of steric factors between the piperazine ring and the attached chlorobenzene ring: the twist for the β polymorph is in the opposite sense to the α and γ polymorphs, but the magnitudes are similar.

Given that the three polymorphs of trazodone dihydrate feature clearly identified and substantial differences in torsion angles about two or more C-C single bonds, we may identify them as ‘true conformational polymorphs’ following the suggestion of Cruz-Cabeza and Bernstein [29] that a difference in torsion angle greater than 95° for a particular bond between polymorphs is required to distinguish conformational polymorphs from ‘ordinary polymorphs.’ Referring to Figure 1 and Table 1, it may be seen that Δε_2_ for the α and β polymorphs is 119.5°, and Δε_3_ for the β and γ polymorphs is 120.5°. These differences in torsion angles of ~120° are satisfyingly close to a simple model of the expected local energy minima about a C-C single bond for its gauche and anti-conformations; we may speculate that in isolation the anti-anti conformation of the β polymorph has the lowest energy of the three polymorphs, but hydrogen bonds and ‘packing forces’ must also be considered when the overall (lattice) energy is considered, and most polymorphs differ in energy by less than 5 kJ mol^−1^ [30].

**Figure 7:**
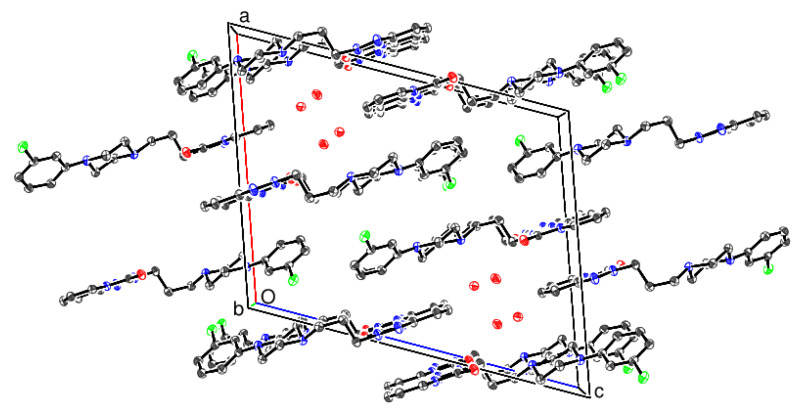
The unit cell packing for β trazodone dihydrate viewed down [010] with H atoms omitted for clarity.

**Figure 8:**
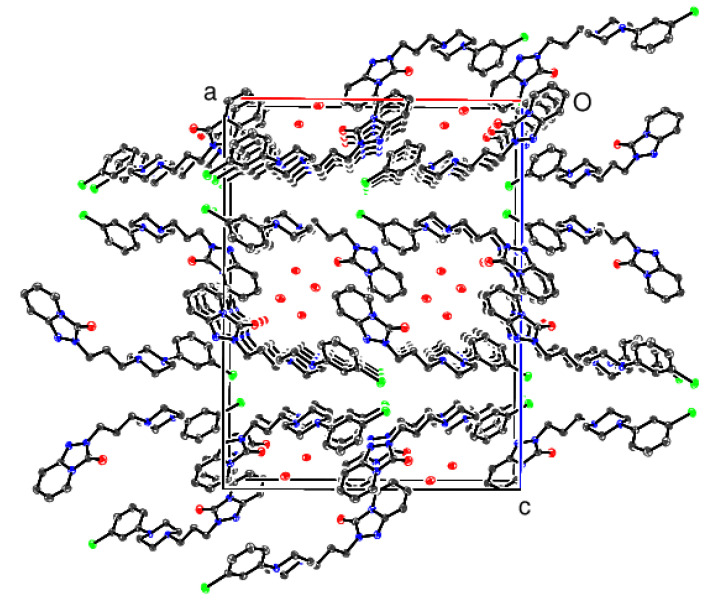
The unit cell packing for γ trazodone dihydrate viewed down [010] with H atoms omitted for clarity.

Views of the crystal packing for the β and γ polymorphs are shown in Figure 7 and Figure 8, respectively. In the β polymorph, the trazodone molecules form pseudo (10$\bar{1}$) layers, which leave small [010] channels occupied by the water molecules, and O-H...O and O-H...N hydrogen bonds (compare Figure 3) generate [010] chains.

In the γ polymorph, the organic molecules form (001) layers, presumably interacting by electrostatic and van der Waals forces. This results in [010] channels occupied by the water molecules of crystallisation (compare Figure 5) with essentially the same O-H...O and O-H...N hydrogen-bonding pattern as the β polymorph.

The α polymorph is affected by severe disorder of both the trazodone molecule and water molecules [24], and not all H atoms could be located, but it appears that the water molecules probably form [100] hydrogen-bonded chains in the extended structure (see supplementary material).

### 2.4. Hirshfeld Surfaces

**Figure 9:**
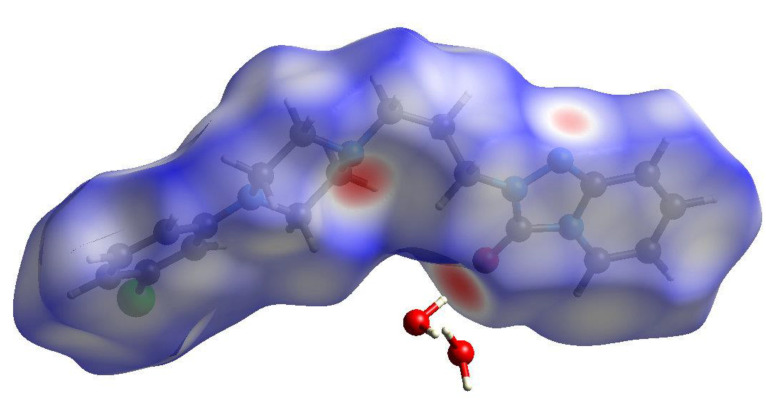
The Hirshfeld surface for β-C_19_H_22_ClN_5_O·2H_2_O mapped over *d*_norm_ (−0.67 to 1.25 arbitrary units).

The Hirshfeld surfaces and two-dimensional fingerprint plots for the β and γ polymorphs were calculated using CrystalExplorer17 [31] using protocols described by Tan et al. [32]. The Hirshfeld surface for the trazodone molecule in β-C_19_H_22_ClN_5_O·2H_2_O mapped over *d*_norm_ is shown in Figure 9: the intense red spots (indicating contacts significantly shorter than van der Waals radii sums) in the vicinity of N4 and O1 correspond to the ‘classical’ hydrogen bonds accepted by these atoms. A somewhat more diffuse spot near N2 corresponds to a weak C-H···N hydrogen bond. There are few indications of red spots in the vicinity of the chlorine atom, which presumably correlates with the long and very weak C-H···Cl interactions associated with C9 and C12.

**Figure 10:**
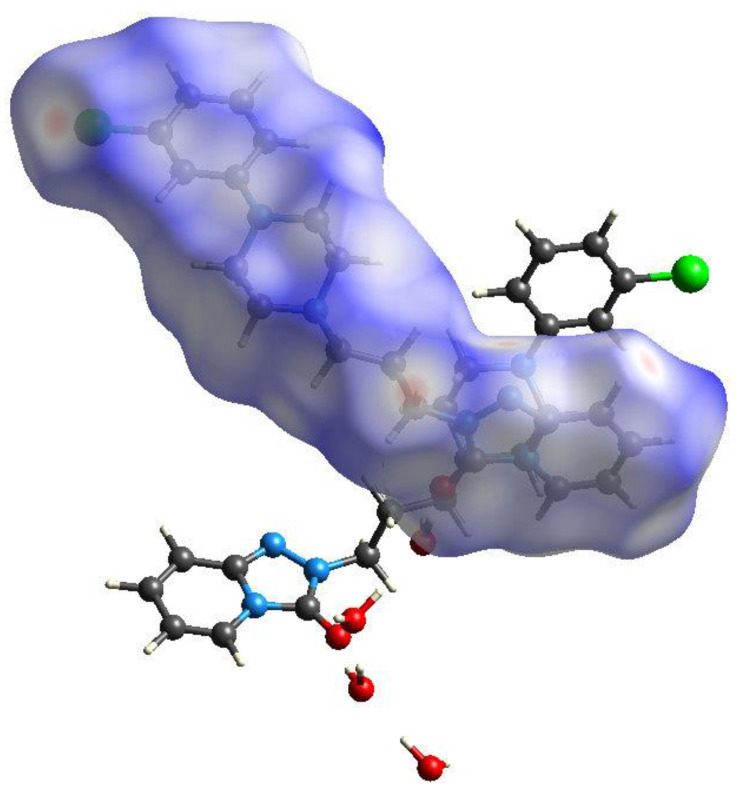
The Hirshfeld surface for the C1 molecules in γ-C_19_H_22_ClN_5_O·2H_2_O mapped over *d*_norm_ (−0.61 to 1.57 arbitrary units).

The Hirshfeld surface for the C1 molecule in γ-C_19_H_22_ClN_5_O·2H_2_O (Figure 10) shows broadly similar features, with intense red spots near O1 and N4 corresponding to the strong hydrogen bonds accepted by these atoms. A faint red spot near Cl1 is somewhat more prominent than the equivalent feature for the Cl atom in the β polymorph. The surface for the C20 molecule (not shown), is very similar in appearance, which is to be expected given the pseudo-symmetry in the structure noted above.

The two-dimensional fingerprint contact percentages for these polymorphs are listed in Table 2. The percentage contribution of H...H contacts is slightly less in the β phase, which is largely made up by a higher percentage of H...C and C...H contacts, and the other contact types are very similar.

## 3. Materials and Methods

A solution of trazodone hydrochloride (100 mg, 0.24 mmol) in warm water (30 mL) was mixed with ammonium carbamate (100 mg, 1.28 mmol) in warm water (30 mL) and left to stand for 2 h. A white product settled to the bottom of the beaker, which was harvested. The powdery starting material was replaced by a microcrystalline phase, and visual inspection indicated that two different crystal morphologies (laths and blocks) had formed.

The crystal structures of β-C_19_H_22_ClN_5_O·2H_2_O (colourless lath 0.24 × 0.09 × 0.02 mm) and γ-C_19_H_22_ClN_5_O·2H_2_O (colourless block 0.27 × 0.22 × 0.12 mm) were established using intensity data collected on a Rigaku AFC11 CCD diffractometer (Cu Kα radiation, λ = 1.54178 Å) at 100 K, and analytical absorption corrections were applied during data reduction (transmission-factor ranges = 0.811–1.00 and 0.559–1.00 for the β and γ polymorphs, respectively). The structures were routinely solved by ‘dual-space’ methods using the program SHELXT [33], and the structural models were completed and optimised by refinement against |*F*|^2^ with SHELXL-2018 [34]. The C-bound H atoms were placed geometrically (C-H = 0.95–0.99 Å) and refined as riding atoms, and the water H atoms were found in difference maps and their positions were freely refined. The constraint *U*_iso_(H) = 1.2 *U*_eq_(carrier) was applied in all cases. Full details of the structures and refinements are available in the deposited cifs.

Crystal data for β-C_19_H_22_ClN_5_O·2H_2_O (C_19_H_26_ClN_5_O_3_): *M*_r_ = 407.90, monoclinic, space group *P*2_1_/*n* (No. 14), *a* = 16.1252 (3) Å, *b* = 6.67160 (9) Å, *c* = 19.8031 (3) Å, β = 108.7862 (19)°, *V* = 2016.94 (6) Å^3^, *Z* = 4, *T* = 100 K, μ = 1.932 mm^−1^, ρ_calc_ = 1.343 g cm^−3^, 12332 reflections measured (6.2 ≤ 2θ ≤ 136.5°), 3658 unique (*R*_int_ = 0.031), *R*(*F*) = 0.033 (3407 reflections with *I* > 2σ(*I*)), *wR*(*F*^2^) = 0.097 (all data), Δρ_min,max_ (*e* Å^−3^) = −0.30, +0.27, CCDC deposition number 2017101.

Crystal data for γ-C_19_H_22_ClN_5_O·2H_2_O (C_19_H_26_ClN_5_O_3_): *M*_r_ = 407.90, orthorhombic, space group *Pca*2_1_ (No. 29), *a* = 20.08500 (16) Å, *b* = 7.62653 (6) Å, *c* = 26.2713 (2) Å, *V* = 4024.21 (5) Å^3^, *Z* = 8, *T* = 100 K, μ = 1.937 mm^−1^, ρ_calc_ = 1.347 g cm^−3^, 16,899 reflections measured (6.7 ≤ 2θ ≤ 136.5°), 6113 unique (*R*_int_ = 0.021), *R*(*F*) = 0.030 (6073 reflections with *I* > 2σ(*I*)), *wR*(*F*^2^) = 0.084 (all data), Δρ_min,max_ (*e* Å^−3^) = −0.26, +0.23, Flack absolute structure parameter = 0.453 (12), CCDC deposition number 2017102.

## 4. Conclusions

In this study, two new dihydrates of trazodone resulted from the same crystallisation reaction, and were characterised by single crystal X-ray structure determinations. Each crystal structure has zigzag chains of hydrogen-bonded water molecules. The propyl chains have different conformations for each structure. The β polymorph has an anti-gauche conformation and the γ polymorph has an anti-anti conformation: the latter is presumed to be the more stable polymorph. The Hirshfeld surfaces show regions of strong hydrogen bonding at the O1 and N4 atoms.

The phenomenon of the spontaneous crystallisation of two polymorphs (one of which must be metastable) from the same solution is an interesting one, and previous reported examples include the formation of α and β L-glutamic acid from aqueous solution [35], and two forms of eflucimibe from the mixed solvents of ethanol and *n*-heptane [36]; a detailed theoretical analysis of the crystallisation thermodynamics and kinetics of the latter system has recently been reported [37]. It is well recognised that temperature and (super) saturation of the solution are key factors in the crystallisation of polymorphs, and we plan to carry out further investigations of the trazodone system to see if the β and γ forms can be made pure and if the α polymorph can be crystallised under different conditions. If the pure polymorphs can be prepared, thermal analysis should provide information on their relative stabilities and dehydration behaviours, which may be combined with quantum chemistry calculations to examine their relative energies.

## Figures and Tables

**Table 1:** Key torsion angles (°) in the polymorphs of C_19_H_22_ClN_5_O·2H_2_O.

Polymorph	ε_1_	ε_2_	ε_3_	ε_4_	ε_5_
α *	−103.45 (19)	56.5 (2)	159.10 (14)	67.89 (19)	32.0 (2)
β	−100.92 (15)	175.98 (11)	−56.11 (16)	−59.16 (14)	−39.09 (17)
γ-C1 molecule	−89.7 (3)	175.8 (2)	−176.6 (2)	−73.1 (3)	45.3 (3)
γ-C20 molecule	−89.1 (3)	175.2 (2)	−177.0 (2)	−73.3 (3)	44.4 (3)

* major disorder component.

**Table 2:** Two-dimensional fingerprint percentage contacts.

Contact Type	β	γ-C1 Molecule	γ-C20 Molecule
H...H	48.7	52.0	52.0
H...C	7.0	3.6	3.7
H...N	2.5	3.1	3.2
H...O	4.5	4.3	4.0
H...Cl	5.3	4.9	5.2
N...H	3.9	4.3	4.3
O...H	4.2	4.4	4.3
C...H	9.4	5.2	5.2
Cl...H	7.5	7.5	7.3

All contacts are ‘inward facing’, i.e., non-reciprocal.

## Data Availability

The crystal structure data are available from the Cambridge Crystallographic Center deposition numbers CCDC 2017101 and CCDC 2017102.

## Supplementary Materials

The following are available online. Table S1: Hydrogen bonds (Å, °) in β-C_19_H_22_ClN_5_O·2H_2_O. Table S2: Hydrogen bonds (Å, °) in γ-C_19_H_22_ClN_5_O·2H_2_O.

## Sample Availability

Samples of the compounds studied are available from the authors.
