# Supplementary material for: Crystalline Forms of Trazodone Dihydrates
Source: Molecules. 2021 Sep 3;26(17):5361. doi: 10.3390/molecules26175361 (PMC8433896; doi:10.3390/molecules26175361)
Supplement: Supplementary file 1 [file molecules-26-05361-s001.zip › molecules-1340603-supplementary.pdf]

Crystalline Forms of Trazodone Dihydrates.

M. John Plater* and William T. A. Harrison
Department of Chemistry, University of Aberdeen, Meston Walk, Aberdeen, AB24 3UE, UK

Supplementary materials

Bond	*D*—*H*	H···*A*	*D*···*A*	*D*—H···*A*
O2—H1w···N4^{i}	0.878 (19)	2.107 (19)	2.8650 (15)	162.2 (17)
O2—H2w···O3^{ii}	0.92 (2)	1.79 (2)	2.7133 (15)	179.5 (17)
O3—H3w···O2	0.90 (2)	1.81 (2)	2.7030 (15)	173.9 (18)
O3—H4w···O1	0.88 (2)	1.85 (2)	2.7303 (14)	175.5 (18)
C1—H1···N2^{iii}	0.95	2.48	3.3911 (18)	161
C4—H4···O2^{iv}	0.95	2.54	3.3922 (17)	149
C9—H9b···Cl1^{v}	0.99	2.90	3.7742 (14)	148
C12—H12b···Cl1^{vi}	0.99	2.93	3.7714 (14)	143

Symmetry codes: (i) 1 ½–*x*, *y*–½, ½–*z*; (ii) 1 ½–*x*, *y*+½, ½–*z*; (iii) *x*, *y*–1, *z*; (iv) 1–*x*, 1–*y*, –*z*; (v) 1–*x*, 1–*y*, 1–*z*; (vi) *x*, *y*+1, *z*.

Table S1 Hydrogen bonds (Å, °) in β-$C_{19}H_{22}ClN_5O·2H_2O$

Bond	*D*—*H*	H···*A*	*D*···*A*	*D*—H···*A*
O3—H1w···O6	0.82 (4)	1.94 (4)	2.747 (3)	169 (4)
O3—H2w···O1	0.92 (4)	1.88 (4)	2.793 (3)	170 (3)
O4—H3w···N4^{i}	0.92 (4)	2.01 (4)	2.923 (3)	171 (3)
O4—H4w···O3	0.77 (4)	1.98 (4)	2.745 (3)	176 (3)
O5—H5w…O4^{ii}	0.85 (4)	1.91 (4)	2.746 (3)	171 (4)
O5—H6w…O2	0.78 (4)	2.02 (4)	2.794 (3)	174 (4)
O6—H7w…N9^{i}	0.84 (4)	2.11 (4)	2.934 (3)	171 (3)
O6—H8w…O5	0.90 (4)	1.84 (4)	2.735 (3)	178 (3)
C4—H4…O4^{iii}	0.95	2.57	3.467 (3)	158
C7—H7a…Cl2^{iv}	0.99	2.71	3.597 (3)	150
C10—H10a…O1^{v}	0.99	2.62	3.373 (3)	133
C17—H17…N2^{vi}	0.95	2.65	3.471 (3)	146
C23—H23…O6^{vii}	0.95	2.58	3.480 (3)	159
C26—H26a…Cl1^{viii}	0.99	2.72	3.659 (3)	159
C29—H29a…O2^{ix}	0.99	2.58	3.329 (3)	132
C36—H36…N8^{x}	0.95	2.61	3.429 (3)	145

Symmetry codes: (i) x, y–1, z; (ii) x, y+1, z; (iii) ½ +x, –y, z; (iv) 1–x, 2–y, ½+z; (v) x–½ , 2–y, z; (vi) x–½ , 1–y, z; (vii) –x , 2–y , z–½ ; (viii) x+½ , 3–y, z.

Table S2 Hydrogen bonds (Å, °) in γ-$C_{19}H_{22}ClN_5O·2H_2O$
